# Clinical Identification and Referral of Adults With Prediabetes to a Diabetes Prevention Program

**DOI:** 10.5888/pcd16.180540

**Published:** 2019-06-27

**Authors:** Christopher S. Holliday, Janet Williams, Vanessa Salcedo, Namratha R. Kandula

**Affiliations:** 1 Improving Health Outcomes, American Medical Association, Chicago, Illinois; 2 Economics and Practice Innovations, American Society of Anesthesiologists, Schaumburg, Illinois; 3 Division of General Internal Medicine and Geriatrics, Northwestern University Feinberg School of Medicine, Chicago, Illinois; 4 Center for Community Health, Institute for Public Health and Medicine, Northwestern University Feinberg School of Medicine, Chicago, Illinois

## Abstract

**Purpose and Objectives:**

Community programs to prevent or delay the onset of type 2 diabetes are effective, but implementing these programs to maximize their reach and impact remains a challenge. The American Medical Association (AMA) partnered with the YMCA of the USA, as part of a Centers for Medicare and Medicaid Innovation demonstration project, to develop, implement, and evaluate innovative quality improvement strategies to increase routine screening, testing, and referral of Medicare patients with prediabetes to diabetes prevention programs (DPPs) at local YMCAs.

**Intervention Approach:**

AMA recruited 26 primary care practices and health systems in 17 US communities to implement point-of-care and retrospective methods (or a combination of both) for screening, testing, and referral of Medicare patients with prediabetes.

**Evaluation Methods:**

We assessed changes in rates of referral and enrollment of patients among participating practices. We used a mixed-methods pretest–posttest evaluation design to determine if use of certain tools and resources, coupled with systems changes, led to increased screening and referrals.

**Results:**

Practices referred a total of 5,640 patients, of whom 1,050 enrolled in a YMCA DPP (19%; range, 2%–98%). Practices (n = 12) that used retrospective (ie, electronic medical record [EMR]) systems to identify eligible Medicare patients via a registry referred more people (n = 4,601) to the YMCA DPP than practices (n = 10) that used a point-of-care method alone (n = 437 patients) or practices (n = 4) that used a combination of these approaches (n = 602 patients). All approaches showed increased enrollment with point-of-care methods being most successful.

**Implications for Public Health:**

Lessons learned from this intervention can be used to increase diabetes prevention in the United States and support the Centers for Medicare and Medicaid Services (CMS) decision to expand Medicare coverage to include the DPP for all Medicare beneficiaries.

**Summary**

**What is already known about this topic?**

The prevalence of type 2 diabetes and prediabetes extends to about one-third of the US adult population. Reducing this burden will require prevention programs, but clinical practices do not routinely screen, test, and refer patients to such programs.

**What is added by this report?**

We describe implementation of diabetes prevention strategies, including robust clinical–community linkages, that helped clinicians and their care teams at 26 health centers to systematically identify patients with prediabetes and refer them to an evidence-based diabetes prevention program.

**What are the implications for public health practice?**

Strategies developed and tested created robust clinical–community linkages that are generalizable across a wide variety of health centers and health systems across the United States.

## Introduction

Type 2 diabetes, a highly prevalent and costly disease in the United States, affects more than 25% of the Medicare population, and its prevalence is projected to increase approximately twofold for all US adults aged 18 to 79 by 2050 if current trends continue (1). An estimated 84 million US adults, about 34% of the population, have prediabetes, but only 12% know they have it (2,3). Almost half (48.3%) of adults aged 65 or older may have prediabetes (2,3). Among those with prediabetes, the risk of developing type 2 diabetes may be 5% to 10% annually and 70% over a lifetime (4). The burden of prediabetes, including its associated risk for heart attack, stroke, and increased medical expenditures, suggests the need for population-based clinical strategies to identify and manage this common metabolic disorder (5). Thus, the US Preventive Services Task Force recommends diabetes screening for adults aged 40 to 70 who are overweight or obese (6). Adherence to this recommendation will identify millions of patients with prediabetes who could benefit from a program to prevent or delay type 2 diabetes.

The landmark 2002 Diabetes Prevention Program, a randomized controlled trial, found that an intensive lifestyle change program focused on diet, physical activity, and weight loss reduced the risk of developing type 2 diabetes by 58% among adults aged 18 or older and by 71% among adults aged 60 or older compared with adults on placebo and that the program was significantly more effective for reducing diabetes risk than metformin (7). The Centers for Medicare and Medicaid Services announced in 2016 that it would begin covering diabetes prevention programs that were part of the Centers for Disease Control and Prevention (CDC) National Diabetes Prevention Program (DPP) for all Medicare beneficiaries beginning in April 2018 (8).

## Purpose and Objectives

US adults make more than 500 million visits to primary care providers annually, making these providers’ offices ideal for identifying patients with prediabetes (9). However, these clinical practices and the health systems that comprise them face barriers to preventive procedures, such as systematic identification and referral of patients with prediabetes to CDC-recognized diabetes prevention programs (10). To maximize the potential of primary care providers to help prevent or delay the onset of type 2 diabetes among the Medicare population, the American Medical Association (AMA) partnered with the YMCA of the USA, as part of a Centers for Medicare and Medicaid Innovation demonstration project, to develop and test innovative quality improvement strategies to implement routine screening, testing, and referral of Medicare patients with prediabetes to DPPs at local YMCAs.

This article describes our evaluation of a pilot systems-change study to integrate screening, testing, and referral of Medicare patients with prediabetes to DPPs. Study findings serve as a framework that can be adopted or adapted to support the Medicare diabetes prevention services that were made available as of April 2018 through the Medicare Diabetes Prevention Program (MDPP) expanded model (11). The study, which was conducted over a 15-month period from 2013 through 2015, was part of a population-based quality improvement strategy in 26 clinical practices and health systems in 8 states that had DPPs in 17 YMCA communities. The assumptions were that 1) clinical practices had no systematic process for screening and testing Medicare patients with prediabetes and referring them to CDC-recognized lifestyle change programs or DPPs, 2) clinical practices that used tailored tools and resources for screening and testing Medicare patients with prediabetes and referral to DPPs would have increased patient referrals and enrollment, and 3) clinical practices would have different numbers of referrals and enrollment of Medicare patients with prediabetes depending on which method of patient identification and which intervention they chose.

## Intervention Approach

We conducted a quasi-experimental, mixed methods, prospective study by using the RE-AIM (reach, efficacy, adoption, implementation, maintenance) implementation science framework (12,13) to determine whether a health system intervention (ie, adoption of a set of tools and resources and health service strategies) in various types of primary care practices increased systematic screening, testing, and referral of Medicare patients with prediabetes to CDC-recognized YMCA DPPs. Registration costs for participants in the year-long program, which averaged $450 per enrollee, were covered under a Centers for Medicare and Medicaid Innovation demonstration grant. Twenty-six clinical practices in 17 US communities were identified by state and county medical societies and the YMCA of the USA on the basis of the presence of a local YMCA with a CDC-recognized DPP within 5 miles of the practice and no previous referrals from that practice to a YMCA DPP. These practices were located in 8 states and varied in size from 2 to 910 physicians. Practices ranged from small, independent practices (generally 10 or fewer physicians), some with multiple sites, to large, integrated health systems (Table 1). The patient population of each practice was not reported because the panel size (patients assigned to a particular provider) varied greatly depending on the referring physician.

**Table 1:** Referral and Enrollment of Medicare Patients in the YMCA’s Diabetes Prevention Program, by Clinical Practice (N = 26) Characteristics, and Methods, March 2014 – June 2015^a^

State	Clinical Site No.	Clinical Practice Type^b^	No. Referring Physicians in Practice	Patient Identification and Intervention Method^c^	No. Patients Referred	No. Patients Enrolled (%)
Delaware	1	Integrated delivery	15	Retrospective + point of care	214	118 (55)
	2	Integrated delivery	3	Point of care	15	10 (67)
	3	Independent	3	Point of care	48	43 (90)
	4	Independent	3	Point of care	2	1 (50)
	5	Independent, multisite	8	Retrospective	589	109 (19)
	6	Independent, multisite	2	Retrospective	277	43 (16)
	7	Independent	6	Retrospective	252	56 (22)
	8	Independent	14	Retrospective	30	6 (20)
	9	Independent	7	Retrospective	40	39 (98)
	10	Independent	8	Retrospective	89	85 (96)
Florida	1	Integrated delivery	4	Retrospective + point of care	93	31 (33)
	2	Independent, multisite	10	Point of care	296	156 (53)
	3	Integrated delivery	3	Retrospective + point of care	16	4 (25)
	4	Integrated delivery	5	Point of care	22	13 (59)
	5	Independent	7	Point of care	4	1 (25)
	6	Independent, multisite	6	Point of care	5	4 (80)
Indiana	1	Integrated delivery	215	Retrospective	200	—d
Minnesota	1	Independent	14	Point of care	30	15 (50)
	2	Integrated delivery	143	Retrospective + point of care	279	156 (56)
New York^e^	1	Integrated delivery	910	Retrospective	2,500	40 (2)
Arizona	1	Integrated delivery	48	Point of care	8	—d
	2	Integrated delivery	6	Point of care	7	—d
	3	Integrated delivery	117	Retrospective	168	20 (12)
Ohio	1	Independent	6	Retrospective^e^	100	—d
	2	Integrated delivery	177	Retrospective	250	100 (40)
Texas	1	Integrated delivery	217	Retrospective	106	—d
Total	26	1,957		5,640		1,050 (19)

a Data were self-reported by practices or reported by YMCAs.

b An integrated delivery system is a network of health care facilities under a parent holding company that provides a continuum of health care services for seamless, coordinated care.  Independent clinics are provider-owned multi-specialty health care clinics guided by the providers who care for their patients. Independent, multisite clinics are provider-owned multi-specialty health care clinics in multiple sites that are guided by the providers who care for their patients.

c Point of care was defined as identifying a patient with prediabetes during an office visit; retrospective was defined as using existing laboratory values in the electronic medical record to create a report or list of patients based on risk factors or laboratory values to identify patients who meet the criteria for prediabetes.

d Data lost to follow-up.

e New York is an outlier with 2,500 referrals. If this site is excluded, retrospective methods still yield more referrals (2,101).

## Evaluation Methods

We chose a mixed-methods pretest–posttest evaluation design to determine if the use of certain tools and resources, coupled with systems changes, led to increased screening and referrals of Medicare patients at high risk for type 2 diabetes to community YMCA DPPs. Measures included pretest and posttest surveys and structured interviews.

We recruited 30 clinical practices in 17 communities to participate in our study; 26 clinics agreed to participate. AMA and YMCA staff members trained physicians and care teams across all practice sites in use of the American Medical Association’s Clinician Diabetes Prevention Toolkit for Identifying Patients with Prediabetes (toolkit) (Table 2). On the basis of feedback obtained from structured interviews with practice staff members, the toolkit was slightly refined for clarity and ease of use before distribution to the 26 clinics. The YMCA DPP intervention consisted of the toolkit, which includes workflows and process maps to identify and refer Medicare patients with prediabetes; direct education for health care teams via in-person trainings and technical assistance; standardized forms for referrals from clinical settings to local DPPs; and pretest and posttest surveys and interviews to determine what worked and what tools needed refinement. These toolkit elements were based on existing models for referring patients to internal medical services (eg, referral to medical nutrition therapy) or to external programs (eg, referral to physical therapy) (14).

**Table 2:** American Medical Association Clinician Diabetes Prevention Toolkit for Identifying Patients with Prediabetes

Tool	Use	How Used
Retrospective algorithm^a^	Querying electronic medical records to identify patients with prediabetes based on HbA_1c_ or glucose levels and BMI (weight in kilograms divided by height in meters squared)	• IT staff codes EMR to develop a list or registry of patients with prediabetes, based on prerecorded HbA_1c_ and BMI values • Practice staff verifies eligibility (HbA_1c_ or glucose level, BMI, and that patient is alive and ambulatory) • Practice staff generates letter to patients informing them that they are at high risk for type 2 diabetes, provides educational materials about prediabetes, and lets the patient know that someone from the YMCA DPP will be contacting them about the program. • Practice staff faxes referral to YMCA DPP for follow-up to enroll patient
Point-of-care method^b^	Identifying patients with prediabetes in office, based on HbA_1c_ or glucose levels and BMI	• Patient completes ADA/CDC paper-based prediabetes risk test (13,14) • Practice staff verifies eligibility (Hb_A1c_ or glucose level, BMI) • Practice staff counsels patient, provides educational materials about prediabetes and the YMCA DPP • Practice staff provides referral to patient and faxes patient information to YMCA DPP for follow-up to enroll patient
Combination of retrospective algorithm and point-of-care method	Applying both methods	Use both retrospective algorithm and point-of-care method concurrently

Abbreviations: ADA, American Diabetes Association; BMI, body mass index; CDC, Centers for Disease Control and Prevention; HbA_1c_, hemoglobin A_1c_; IT, information technology; YMCA DPP, YMCA’s Diabetes Prevention Program.

**Figure 1:**
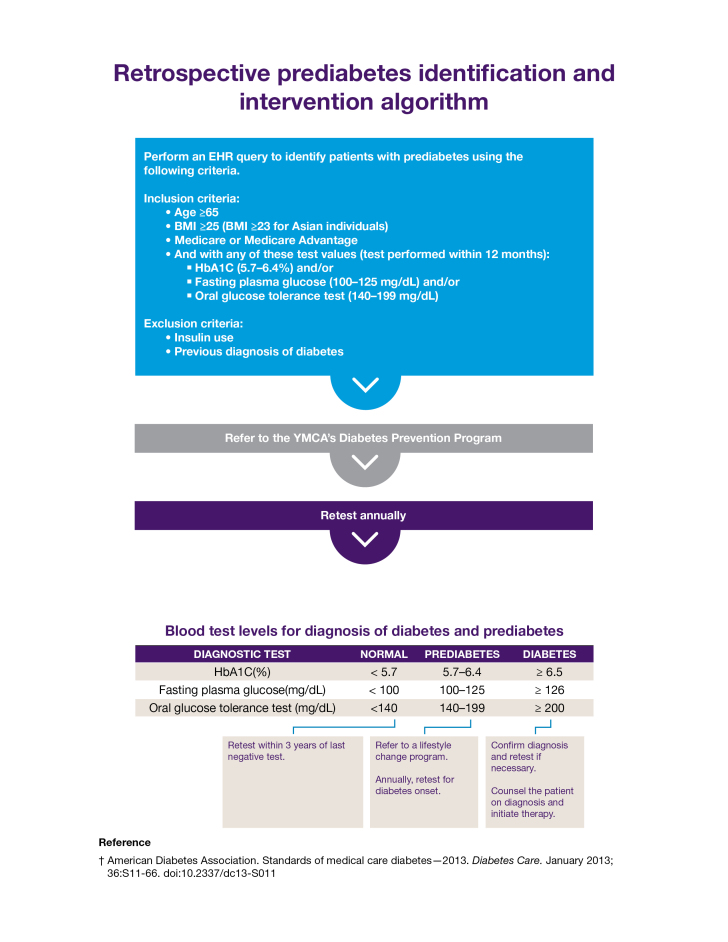
Handout for clinical practices used in YMCA’s Diabetes Prevention Program showing the retrospective prediabetes identification and intervention algorithm developed by the American Medical Association to identify patients with prediabetes for referral to the program. Abbreviations: BMI, body mass index; EHR, electronic health record; HbA1C, hemoglobin A1c. Reprinted with permission of the American Medical Association.

**Figure 2:**
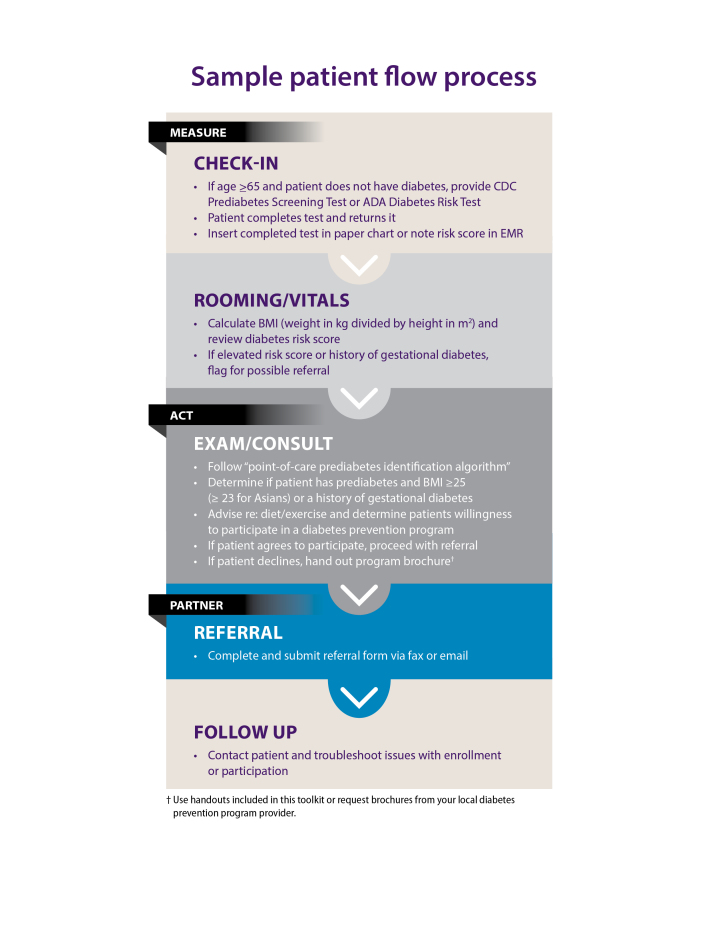
Handout for clinical practices used in the YMCA’s Diabetes Prevention Program showing a patient workflow process using point-of-care methods to identify candidates for referral to the program. Abbreviations: ADA, American Diabetes Association; CDC, BMI, body mass index; Centers for Disease Control and Prevention; EMR, electronic medical record; GDM, gestational diabetes mellitus. Reprinted with permission of the American Medical Association.

a Illustrated by Figure 1.

b Illustrated by Figure 2.

The toolkit included a retrospective algorithm (Figure 1) for querying electronic medical records (EMRs) to identify patients with prediabetes on the basis of a hemoglobin A_1c_ value of 5.7% to 6.4% or fasting plasma glucose levels of 100 to 125 mg/dL and a body mass index (BMI, weight in kg divided by height in m^2^) of 25 or more. The toolkit recommended verifying blood glucose levels in the prediabetes range and provided criteria for referring patients to YMCA DPPs. The toolkit also included point-of-care methods to identify candidates for YMCA DPP referral. Practices integrated a prediabetes screening and referral process workflow (Figure 2) into their daily patient care. The University of Illinois at Chicago Institutional Review Board reviewed the study (Diabetes Prevention Physician Referral Program, protocol no. 2013–1258) and exempted it from full review.

**Table:** 

Values	Diagnostic Test		
	HbA_1c_	Fasting Plasma Glucose, mg/dL	Oral Glucose Tolerance Test, mg/dL
Normal	<5.7	<100	<140
Prediabetes	5.7–6.4	100–125	140–199
Diabetes	≥6.5	≥126	≥200
Action	Retest within 3 years of last negative test	Refer to a lifestyle change program; annually, retest for diabetes onset	Confirm diagnosis and retest if necessary; counsel the patient on diagnosis and initiate therapy

The study was conducted from March 2013 through June 2015. Data collection on patients referred to the program began in March 2014. Each practice was trained on the refined toolkit (Table 2) and each chose a strategy or approach based on its staffing resources. Practice staff members (ie, medical assistants, clerical staff) asked Medicare patients to complete 1 of 2 screening tests (15,16) to identify prediabetes risk. If a patient was at risk, the clinical staff (physician, nurse) verified eligibility and determined if a referral to a YMCA DPP was appropriate. As part of the referral, primary care providers briefly counseled patients and provided an educational handout about prediabetes and the YMCA DPP.

In lieu of point-of-care screening and referral methods, several clinical practices used the retrospective (EMR) algorithm (Figure 1) to query their electronic records to create a prediabetes registry. A subset of practices generated a prediabetes registry as well as integrated screening and referral at the point of care. The practice staff contacted patients from the registry via telephone, email, letter, or postcard to explain what prediabetes was and how it increased the risk of type 2 diabetes and to encourage patients to participate in a YMCA DPP that was designated by YMCA of the USA. A business associate agreement between the local YMCA and the clinical practice allowed these practices to provide information on eligible patients to the local YMCA DPP coordinator and for that coordinator to record and report the number of patients referred and enrolled in their YMCA DPP each month. The agreement assured the safe exchange of protected health information in accordance with HIPAA (Health Insurance Portability and Accountability Act) guidelines. Once the YMCA DPP coordinator received the registry list of referrals or each of the point-of-care referrals, that person contacted patients to enroll them. Enrollment was confirmed when a patient registered and attended the first class. Concurrently, the clinical practice staff flagged patients’ medical records with a reminder to physicians to discuss program participation with patients at the next office visit. If a patient declined to participate, physicians at follow-up discussed the importance of lifestyle change for diabetes prevention and encouraged enrollment in a YMCA DPP.

This implementation evaluation was designed by using the RE-AIM (reach, efficacy, adoption, implementation, maintenance) implementation science framework (12) to assess whether the adoption of a set of tools and resources and health service strategies increased systematic screening, testing, and referral of Medicare patients with prediabetes to CDC-recognized YMCA DPPs. The YMCA DPPs selected were part of the CDC National DPP recognition program, and used the standardized curriculum, although they may not have achieved full recognition at the time of our pilot study. By using the RE-AIM implementation science framework, the impact of the intervention was determined as a function of the framework’s 5 factors (reach, efficacy, adoption, implementation, maintenance) (Box).

**Box. RE-AIM (Reach, Efficacy, Adoption, Implementation, Maintenance) Model As Applied to Clinician Referrals to YMCA’s Diabetes Prevention Program**

**Table:** 

Component	Study Factors Description
Reach	Number of at-risk patients identified, number of referrals made, number enrolled, and proportion of the referred that enrolled
Efficacy	Number of at-risk patients identified, number of referrals made, number enrolled, and proportion of patients referred who enrolled from baseline, as a function of the method(s) used for screening, testing, and referring adult Medicare patients with prediabetes
Adoption	Proportion and representativeness of clinical settings that adopt point-of-care, retrospective, or a combination of both methods for screening, testing, and referring adult Medicare patients with prediabetes
Implementation	Implementation of point-of-care, retrospective, or a combination of both methods for screening, testing, and referring adult Medicare patients with prediabetes
Maintenance	Extent to which implementation of point-of-care, retrospective, or a combination of both methods for screening, testing, and referring adult Medicare patients with prediabetes is preferred and maintained or repeated

## Evaluation

To better understand reach and efficacy, a 13-item pretest survey was administered online that asked about practice type, existing screening and referral practices, and the demographics of the clinical practice setting (ie, location, system type, specialty). The survey also asked practices to identify facilitators and barriers to the use of workflows and algorithms and asked about attitudes and behaviors among practice clinicians regarding prediabetes (eg, Does your practice refer patients with prediabetes to community programs for lifestyle interventions?). This quantitative pretest survey was distributed to multiple clinic staff members (eg, physicians, nurses, medical assistants, physician assistants) and was completed before beginning the pilot study.

The same 13-item online survey was administered at the start of the pilot and at the end as a posttest survey of the same clinic staff members to measure changes in attitudes and behaviors regarding prediabetes screening, testing, and referral. The survey also contained a question on adoption of the toolkit. Practices worked with the YMCA DPP to track the number of at-risk patients identified and referred, number enrolled, and proportion of patients referred who enrolled. Each clinical site was given a form that could be updated electronically with the number of Medicare patients with prediabetes referred each month. These referrals were compared with the referral and enrollment numbers captured by the associated YMCA DPP. Patient demographic characteristics were not collected, to reduce burden of reporting for each site.

The qualitative assessment included semistructured interviews conducted with clinical practice staff members to identify health care system barriers to screening and referral strategies and to better understand changes in adoption, implementation, and maintenance. The interviews were conducted per practice by telephone and were recorded and transcribed verbatim. The transcripts were analyzed by using NVivo qualitative data analysis software (QSR International), which categorized and classified that qualitative data into themes and attributes.

## Results

Results were analyzed on each of the associated RE-AIM study factors.

**Reach.** The 26 participating clinical practices moved from no referrals of Medicare patients to referral of 5,640 Medicare patients with prediabetes to the YMCA’s DPP (Table 1). All clinical practices referred patients, and all had increased enrollment from baseline. Pretest and posttest awareness and behavior revealed important changes in clinical behavior. Across the clinical practice sites, pretest surveys (n = 48) and posttest surveys (n = 44) were most often completed by primary care physicians (67%–72%), followed by nurses (14%–15%), nurse practitioners (7%–15%), medical assistants (2%), physician assistants (2%), health educators (2%), receptionists (2%), and social workers (2%). The same staff member completed both pretest and posttest surveys. Findings indicate that knowledge about prediabetes and routine screening levels for the condition was high among referring physicians and care team members in both the pretest and posttest surveys. The pretest survey showed that 59% of clinical practice staff members agreed or strongly agreed that they were aware of community resources that help patients prevent diabetes, and 84% were aware in the posttest survey. In the pretest survey, 53% of physicians said they agreed or strongly agreed that they referred patients with prediabetes to community resources that help prevent diabetes, and in the posttest survey that increased to 83% of physicians. Referral rates did, however, differ on the basis of the method used by the practice. Slightly more clinical settings (n = 16) chose to use the retrospective method to develop a registry of their Medicare patients with prediabetes rather than the point-of-care method (n = 14). The 12 practices that used only a retrospective method referred a greater number of Medicare patients (n = 4,601) than the 10 practices that used only a point-of-care method (n = 437) or 4 practices that used a combination of these methods (n = 602).

**Efficacy.** Of the 5,640 Medicare patients referred to a YMCA DPP, 1,050 (19%) enrolled. Enrollment rates varied widely across clinical sites, ranging from 2% to 98%. The weighted average for enrollment across all sites was 49%. The highest enrollment rates (90%, 96%, and 98%) were from independent clinical practices. Practices that used only a retrospective method had a lower rate of enrollment (11%) than those that used only the point-of-care method (56%) and those that used a combination of retrospective and point-of-care methods (51%). Although the point-of-care method had the highest enrollment rate (56%), that method had the lowest number of referrals. A small proportion of sites (19%) that used retrospective or point-of-care methods only had referrals, but no subsequent enrollment.

**Adoption.** Structured interviews with clinical staff members (n = 44) in 26 clinical practices revealed that point-of-care, retrospective, or combination strategies were uniformly adopted at each site as intended, depending on which strategy clinical teams chose. The 26 clinical practice sites varied in size from 2 to more than several hundred physicians. Half (n = 13, 50%) were small, independent practices, 4 of which had multiple sites. The other half was made up of large, integrated health systems. The practices also varied in geographic distribution across the East, Midwest, and South and in size of patient population. All referring physicians were primary care providers (ie, family medicine) and further demographic data were not collected. No differences were reported or observed among the physicians. Some physicians were informed of their patients with prediabetes as a result of the generation of a patient registry by clinic staff. Physicians were consulted by clinic staff to verify prediabetes and to approve the referral.

**Implementation.** We saw no preference of strategy implemented that was based on clinical setting type, although slightly more integrated delivery sites chose the retrospective strategy, probably a result of ease of registry development within their EMR systems. Clinical staff members reported that it was helpful to frame screening and referral as a quality improvement strategy rather than an additional requirement and that screening and referral could be operationalized across various team members, with any one team member being the lead or champion. The clinical staff also identified barriers to implementing screening and referral strategies. Staff members reported challenges, such as not having enough staff members to query the EMR to identify Medicare patients at risk for prediabetes and to create a prediabetes registry. Staff members were also uncertain about the best ways to integrate identification and referral into busy clinical workflows at the point of care. The staff had concerns about additional work load and sustainability; staff members spoke specifically about how to continue to screen, test, and refer patients and maintain behavior change when patients and providers faced competing medical problems and priorities. Unique contextual factors, such as patient readiness for change and YMCA DPP program accessibility, were also mentioned as important factors that affected implementation. At a few sites, strategies were not sufficiently implemented because of various factors, including having no one available to code the retrospective algorithm in the EMR system or because enrollment data from YMCA DPPs were missing or lost to follow-up.

**Maintenance.** More than a third of practices (n = 10) reported that they continued to use AMA referral tools in their practice at 6 months beyond the pilot. Practices preferred using retrospective identification of Medicare patients when dedicated staff members were available to run queries and maintain a registry to identify patients with prediabetes.

## Implications for Public Health

Despite the availability of effective, community-based YMCA DPPs (17,18), a gap remains between identification and referral of Medicare patients with prediabetes to lifestyle change programs (18). The results of this study can help accelerate translation of evidence into real-world clinical settings, particularly as the results relate to the identification and referral of Medicare populations at high risk for type 2 diabetes, a subset of the nearly 84 million US adults with prediabetes.

This implementation evaluation revealed that increased awareness and simple modifications to clinical workflows led to increased screening and referrals to YMCA DPPs for preventing type 2 diabetes. Before engaging in this effort, the identified clinical practices were not screening Medicare patients for prediabetes or referring patients with prediabetes to evidence-based lifestyle change programs. Because of the intervention, during a 15-month period the 26 participating clinical practices and health systems began routinely screening patients suspected of having prediabetes, confirming prediabetes by blood test, and referring patients to YMCA DPPs.

Key lessons learned were that framing screening and referral as a quality improvement strategy rather than an additional requirement resulted in greater engagement by busy clinicians. Diabetes prevention is a team sport, and collective buy-in through team-based care is essential. A practice champion is needed but does not have to be a physician.

Only integrated delivery systems practices chose the combination approach, likely because of their higher capacity. Practices preferred using retrospective identification when dedicated staff members were available to run queries and maintain a registry that could be used to identify patients with prediabetes. Referral to a YMCA DPP can be integrated into existing referral systems used by clinical practices, such as those for referring to a physical therapist or dietitian–nutritionist. Opportunities to build screening and referral models as part of value-based care include tying payment incentives, prediabetes screening, and referral into annual check-ups, which can increase the probability that diabetes prevention becomes part of routine care.

The highest numbers of referrals were from health systems or clinical practices that used retrospective methods to query their EMRs to create a prediabetes registry. This approach ensured that Medicare patients with prediabetes were identified, an opportunity that is often missed during an acute or routine visit when competing priorities exist. More referring physicians are captured with the retrospective method because this is a systems approach to identifying patients within the EMR across multiple physician panels.

Physician referrals done at the point of care seemed to yield a higher enrollment rate. Although the retrospective method generated more referrals by volume, it did not yield as many enrollments as the other methods. Although fewer clinical sites chose deploying both methods concurrently over deploying only one method, they experienced more referrals than clinical sites that used the point-of-care method alone and a comparable enrollment rate. Small, independent clinical practices had the highest percentage of patients who enrolled in a YMCA DPP. Those practices had a smaller patient population and strong physician–patient relationships. Clinical settings that used AMA tools to deploy a combination of retrospective and point-of-care methods to identify their Medicare patients with prediabetes increased screening, testing, and referral of these patients to CDC-recognized lifestyle change programs. The capacity of a health system or clinical practice to deploy both methods concurrently is an important consideration. However, physician involvement at the point of care increased rates of enrollment. Some studies suggest that physician recommendation and discussion can increase patient motivation to change certain behaviors, including diet, physical activity, and weight loss.

Our study had limitations. The study was a convenience sample of clinical practices and YMCA DPPs. A primary limitation was that we did not determine the total number of patients served by each of the clinical practices; therefore, the proportion of Medicare patients screened to those referred cannot be determined. In addition, the number of referred Medicare patients was low when considering the average referral rate by practice or by provider — in some cases fewer than 5 referrals per provider over the course of the pilot study. Also, for a small number of clinical sites that used retrospective or point-of-care methods only, referrals were made, but no record was kept of enrollment. Some physicians reported referral of Medicare patients at the outset of the pilot, but no referrals or enrollment could be verified. Overreporting of referrals of Medicare patients with prediabetes by physicians before the pilot study may have been due to social desirability bias. Lower enrollment may have been due to communications issues between the clinical practice and the local YMCA DPP or between provider and patient or to other factors that prohibited conversion of referrals to enrollment (eg, patient readiness). The missing enrollment numbers were patients considered lost to follow-up after initial enrollment.

Community-based organizations such as local YMCA DPPs are promising channels for wide-scale dissemination of low-cost approaches to lifestyle changes for diabetes prevention. Our study found that primary care is a potentially ideal setting for routinely screening and testing Medicare patients for prediabetes and then referring them to a YMCA DPP; a robust linkage between the 2 settings is an effective way to prevent type 2 diabetes. As a next step, AMA is working with national, state, and community partners to implement and scale these strategies in diverse health care delivery systems with the goal of reducing the burden of diabetes in the United States. In addition, AMA will be developing a physician-focused educational module on the Medicare Diabetes Prevention Program. The goal is to ensure all that program-eligible Medicare beneficiaries are referred by their primary care physician to an MDPP.

Developing and testing strategies that operationalize a linkage between the clinical setting and community resources can improve the capacity of the US health care system to respond to the 84 million Americans with prediabetes. Learnings from this study and the strategies tested are generalizable in a wide variety of health centers and health systems across the United States. Our findings can also have an impact, because the approach described in this article can be disseminated and implemented in clinics and communities in need of population health approaches to type 2 diabetes prevention and can be adapted to support the new set of covered services made available in 2018 through the CMS MDPP Expanded Model.

## References

[1] Centers for Disease Control and Prevention. Diabetes Prevention Recognition Program: Working with Medicare beneficiaries guide for CDC-recognized organizations. https://www.cdc.gov/diabetes/prevention/pdf/ta/Implementation-Guide-Medicare.pdf. Accessed December 30, 2018.

[2] Centers for Disease Control and Prevention. National diabetes statistics report, 2017. Atlanta (GA): Centers for Disease Control and Prevention, US Department of Health and Human Services; 2017.

[3] Centers for Disease Control and Prevention. National diabetes statistics report, 2017: estimates of diabetes and its burden in the United States. https://www.cdc.gov/diabetes/pdfs/data/statistics/national-diabetes-statistics-report.pdf. Accessed September 27, 2018.

[4] Tabák AG , Herder C , Rathmann W , Brunner EJ , Kivimäki M . Prediabetes: a high-risk state for diabetes development. Lancet 2012;379(9833):2279–90. 10.1016/S0140-6736(12)60283-9 22683128 PMC3891203

[5] Dall TM , Yang W , Halder P , Pang B , Massoudi M , Wintfeld N , The economic burden of elevated blood glucose levels in 2012: diagnosed and undiagnosed diabetes, gestational diabetes mellitus, and prediabetes. Diabetes Care 2014;37(12):3172–9. 10.2337/dc14-1036 25414388

[6] Siu AL ; US Preventive Services Task Force. Screening for abnormal blood glucose and type 2 diabetes mellitus: US Preventive Services Task Force recommendation statement. Ann Intern Med 2015;163(11):861–8. 10.7326/M15-2345 26501513

[7] Knowler WC , Barrett-Connor E , Fowler SE , Hamman RF , Lachin JM , Walker EA , ; Diabetes Prevention Program Research Group. Reduction in the incidence of type 2 diabetes with lifestyle intervention or metformin. N Engl J Med 2002;346(6):393–403. 10.1056/NEJMoa012512 11832527 PMC1370926

[8] Centers for Medicare & Medicaid Services (CMS), HHS. Medicare program; revisions to payment policies under the physician fee schedule and other revisions to Part B for CY 2018; Medicare shared savings program requirements; and Medicare Diabetes Prevention Program. Final Rule. Fed Regist 2017;82(219):52976–3371. 29231695

[9] Centers for Disease Control and Prevention. Physician office visits, by selected physician characteristics: 2010 National Ambulatory Medical Care Survey. Atlanta (GA): Centers for Disease Control and Prevention/National Center for Health Statistics; 2010.

[10] Gowin E , Dytfeld J , Michalak M , Horst-Sikorska W . Barriers in the delivery of preventive procedures in primary health care. Fam Med Med Sci Res 2012;1(1):101. 10.5114/aoms.2012.30294 PMC3460507 23056084

[11] Balk EM , Earley A , Raman G , Avendano EA , Pittas AG , Remington PL . Combined diet and physical activity promotion programs to prevent type 2 diabetes among persons at increased risk: a systematic review for the Community Preventive Services Task Force. Ann Intern Med 2015;163(6):437–51. 10.7326/M15-0452 26167912 PMC4692590

[12] What is RE-AIM. http://www.re-aim.org/about/what-is-re-aim/. Accessed April 19, 2019.

[13] Glasgow RE , Vogt TM , Boles SM . Evaluating the public health impact of health promotion interventions: the RE-AIM framework. Am J Public Health 1999;89(9):1322–7. 10.2105/AJPH.89.9.1322 10474547 PMC1508772

[14] Centers for Disease Control and Prevention. How to quit smoking. https://www.cdc.gov/tobacco/campaign/tips/quit-smoking/. Accessed September 28, 2018.

[15] American Diabetes Association. Type 2 diabetes risk test. http://www.diabetes.org/are-you-at-risk/diabetes-risk-test/. Accessed September 27, 2018.

[16] Centers for Disease Control and Prevention. CDC prediabetes screening test. https://www.cdc.gov/diabetes/prevention/pdf/prediabetestest.pdf. Accessed September 27, 2018.

[17] Ackermann RT , Finch EA , Brizendine E , Zhou H , Marrero DG . Translating the Diabetes Prevention Program into the community. The DEPLOY pilot study. Am J Prev Med 2008;35(4):357–63. 10.1016/j.amepre.2008.06.035 18779029 PMC2610485

[18] Ackerman RT , Finch EA , Caffrey HM , Lipscomb ER , Hays LM , Saha C . Long term effects of a community-based lifestyle intervention to prevent Type 2 diabetes: the DEPLOY extension pilot study. Chronic Illn 2011;7(4):279–90. 10.1177/1742395311407532 21840914

